# Biocompatible Materials Based on Self-Assembling Peptides on Ti25Nb10Zr Alloy: Molecular Structure and Organization Investigated by Synchrotron Radiation Induced Techniques

**DOI:** 10.3390/nano8030148

**Published:** 2018-03-07

**Authors:** Valeria Secchi, Stefano Franchi, Marta Santi, Alina Vladescu, Mariana Braic, Tomáš Skála, Jaroslava Nováková, Monica Dettin, Annj Zamuner, Giovanna Iucci, Chiara Battocchio

**Affiliations:** 1 Department of Science, Roma Tre University of Rome, Via della Vasca Navale 79, 00146 Rome, Italy; valeria.secchi@uniroma3.it (V.S.); santimarta3@gmail.com (M.S.); giovanna.iucci@uniroma3.it (G.I.); 2 National Institute for Optoelectronics, 409 Atomistilor St., 077125 Magurele, Romania; alinava@inoe.ro (A.V.); mariana.braic@inoe.ro (M.B.); 3 Department of Surface and Plasma Science, Faculty of Mathematics and Physics, Charles University, V Holešovičkách 2, 18000 Prague, Czech Republic; tomas.skala@elettra.eu (T.S.); jaroslava.lavkova@gmail.com (J.N.); 4 Department of Industrial Engineering, University of Padua, Via Marzolo, 9, Padua 35131, Italy; monica.dettin@unipd.it (M.D.); annj.zamuner@studenti.unipd.it (A.Z.)

**Keywords:** synchrotron radiation induced spectroscopies, XPS, NEXAFS, nanostructures, titanium alloy, self-assembling peptides, bioactive materials

## Abstract

In this work, we applied advanced Synchrotron Radiation (SR) induced techniques to the study of the chemisorption of the Self Assembling Peptide EAbuK16, i.e., H-Abu-Glu-Abu-Glu-Abu-Lys-Abu-Lys-Abu-Glu-Abu-Glu-Abu-Lys-Abu-Lys-NH_2_ that is able to spontaneously aggregate in anti-parallel β-sheet conformation, onto annealed Ti25Nb10Zr alloy surfaces. This synthetic amphiphilic oligopeptide is a good candidate to mimic extracellular matrix for bone prosthesis, since its β-sheets stack onto each other in a multilayer oriented nanostructure with internal pores of 5–200 nm size. To prepare the biomimetic material, Ti25Nb10Zr discs were treated with aqueous solutions of EAbuK16 at different pH values. Here we present the results achieved by performing SR-induced X-ray Photoelectron Spectroscopy (SR-XPS), angle-dependent Near Edge X-ray Absorption Fine Structure (NEXAFS) spectroscopy, FESEM and AFM imaging on Ti25Nb10Zr discs after incubation with self-assembling peptide solution at five different pH values, selected deliberately to investigate the best conditions for peptide immobilization.

## 1. Introduction

The increased interest in titanium (Ti) and its alloys for dental implants and prosthesis application derives from their exceptional mechanical properties, corrosion resistance and biocompatibility [1,2]. A gentle surgical technique combined with sufficient healing time has long been considered the key to osseo-integration, and excellent long-term clinical outcome for dental implant thus validates the results of pre-clinical experimental studies. Alloying improves the mechanical properties of titanium for use in high load-bearing applications, total hip, and total knee replacements. However, some concerns related to the toxicity of various alloying elements do exist [3]. In particular, Ti6Al4V alloy is commonly used in clinical practice as biocompatible material for prosthetics applications and dental implants [4,5,6,7,8,9]. In the last years, the in vitro and in vivo tests performed on Ti6Al4V alloy showed that this alloy has a toxic effect resulting from released V and Al and that its elastic modulus is very distant from the bone value [10,11,12,13,14,15,16], restricting its use in biomaterial applications. On this basis, a lot of experiments have been carried out to develop a novel Ti based alloy consisting only of biocompatible elements, which could replace the Ti6Al4V alloy in clinical practice [9,17,18,19]. For example, Ti6Al7Nb (ASTM F1295), Ti13Nb13Zr (ASTM F1713), and Ti12Mo6Zr (ASTM F1813) were proposed as candidates for manufacturing surgical implants. It is worth mentioning that there are a lot of other proposed alloys in the literature, such as Ti-Nb-Zr-Ta [20,21,22,23,24], Ti-Mo-Zr-Fe [25,26], Ti-Al-Zr [27], Ti-Al-Fe [18], Ti-Nb-Fe [28,29], Ti-Nb-Zr-Sn [30] and Ti-Nb [31] systems, but no standards have been published.

The here reported Ti-Zr-Nb system was selected for the following reasons: all of the constituent elements are considered to be highly biocompatible [32,33,34] and show high affinity to oxygen, leading to the formation of stable oxides which improve the corrosion resistance [35,36,37,38,39]; moreover, Zr is added in the alloy due to its capacity to stabilize the β phase. In fact, Abdel-Hady et al. showed that a Zr content ranging from 6 to 30 at % stabilized the β phase in alloys [40]. In the literature, there are few papers dealing with the effect of Zr content on the mechanical, tribological, and anticorrosive properties of Ti-Zr-Nb systems used for biomedical applications. In all published literature, the Zr content is up to 10 at % [41,42,43,44,45,46] or of about 30 at % [43]. Nb addition is also required because it maintains the β phase formed during the annealing. Furthermore a possible strategy to promote osseo-integration and enhance the biological acceptance of the implants is the biofunctionalization of the Ti25Nb10Zr surface with bioactive molecules that can be grafted on the surface in order to establish a molecular dialogue with host cells [47]. Among other bioactive molecules, self-assembling peptides (SAPs) are extremely promising candidates, since thanks to their on-purpose designed sequence they are able to self-assemble in a beta-sheet secondary structure [48,49]. They can then aggregate in the presence of saline creating hydrogels that can be used either as drug delivery vehicles, in the case of factors to release with a precise kinetic, or can be decorated with adhesive sequences or proteins, appropriately conjugated with a self-assembling sequence, allowing the functionalization of the scaffold with adhesive signals in a 3D structure by simple co-aggregation. The chemically stable SAP adhesion to the substrate is usually obtained by covalently and selectively functionalizing the alloy surface of the ion complementary peptide [50,51].

In this work, we present the characterization, carried out by synchrotron radiation-induced X-ray Photoemission Spectroscopy (SR-XPS), angle-dependent Near Edge X-rays Absorption Fine Structure (NEXAFS) spectroscopy, Field Emission Scanning Electron Microscopy (FE-SEM) and Atomic Force Microscopy (AFM) investigations of Ti25Nb10Zr alloy surfaces functionalized by the SAP EAbuK16 (Abu stands for α-aminobutyric acid), i.e., H-Abu-Glu-Abu-Glu-Abu-Lys-Abu-Lys-Abu-Glu-Abu-Glu-Abu-Lys-Abu-Lys-NH_2_. The proposed SAP is able to self-assemble in aqueous solution in the presence of monovalent cations. To prepare the material, Ti25Nb10Zr discs were exposed to self-assembling peptide solutions at pH values ranging from 2 to 12, in order to understand the best conditions for peptide immobilization.

## 2. Materials and Methods

### 2.1. Samples Preparation

#### 2.1.1. Ti25Nb10Zr Alloy Preparation and Preliminary Characterization

Ti25Nb10Zr (in wt %) was manufactured by Romanian Company (R&D Consulting and Services, Bucharest, Romania). Ti25Nb10Zr alloy was casted by a cold crucible levitation melting technique (CCLM), using a FIVES CELES—CELLES MP 25 furnace with nominal power 25 kW (Fives Celes, Lautenbach, France). The alloy was produced by mixing ultra-pure raw metals, subsequently annealed at 900 °C for 5 h in an oven (Caloris-CD 1121) (Caloris, Bucharest, Romania) and cooled in air. For this study, the alloy was cut as discs by a turning machine and mid-polished using 3 µm diamond emery paste.

The elemental composition and distribution of each constituent element of the alloy was checked by means of energy-dispersive X-ray spectroscopy (EDS), using the X-ray detector (EDS-Quantax70, Bruker, Billerica, MA, USA) attached to a scanning electron microscope (SEM, Hitachi TM3030PLUS) (Hitachi, Tokyo, Japan). The compositional analysis was performed automatically by the Quantax 70 microanalysis software (Bruker). The surface morphology of the Ti25Nb10Zr alloy substrates was investigated by atomic force microscopy (AFM) and scanning electron microscopy (SEM). AFM measurements were performed in tapping mode on 30 × 30 µm^2^ area using an INNOVA microscope (Veeco, Plainview, NY, USA). The crystallographic structure was analyzed by X-ray diffraction (XRD) (Rigaku, Tokyo, Japan) using a diffractometer SmartLab Rigaku in the 2θ range 20–100°. The step Δ2θ was 0.02, and the minimum speed was 0.0002 deg/min. The CuKα radiation was used with a wavelength of λ = 1.5411 Å at 45 kV high voltage and 200 mA of the X-ray tube.

Since the alloy is prepared with the aim to be used as material for orthopaedic implants, special attention was devoted to the evaluation of the corrosion resistance in two solutions mimicking the physiological conditions: simulated body solution (SBF, composition: 8.035 g/L NaCl, 0.335 g/L NaHCO_3_, 0.225 g/L KCl, 0.231 g/L K_2_HPO_4_∙3H_2_O, 0.311 g/L MgCl_2_∙6H_2_O, 0.292 g/L CaCl_2_, 0.072 g/L Na_2_SO_4_, 6.228 g/L Tris-(HOCH_2_)_3_CNH_2_ [52]) and Hank solution (composition: 8 g/L NaCl, 0.4 g/L KCl, 0.1 g/L MgCl_2_∙6H_2_O, 0.14 g/L CaCl_2_, 1 g/L glucose, 0.35 g/L NaHCO_3_, 0.06 g/L NaH_2_PO_4_·6H_2_O, 0.06 g/L KH_2_PO_4_, 0.06 g/L MgSO_4_ [53]). The corrosion resistance was evaluated by potentiodynamic polarization tests at 37 ± 0.4 °C using a VersaSTAT 3 Potentiostat/Galvanostat (Princeton Applied Research-AMETEK, Oak Ridge, TN, USA), following the steps:

Monitoring the open circuit potential (OCP) for 15 h after the immersion in electrolyte.

Plotting potentiodynamic curves −2 V vs. OCP to 2 V vs. SCE.

A conventional three-electrode cell was used, with a saturated calomel electrode (SCE) as reference, a platinum one as counter electrode, and the sample (1 cm^2^) as working electrode. For the tests, the scanning rate was of 1 mV/s, value recommended also by ASTM G 59–97. During the tests, the solution was agitated by magnetic stirrer at 150 rpm for elimination of the gas bubbles formed during the test.

On the basis of potentiodynamic curves, both the corrosion potential (*E*_i=0_) and corrosion current density (*i*_corr_) were estimated. The polarization resistance (*R*_p_) was calculated using the Stern–Geary Equation (1) [54]:

(1) $$R_{p}=\frac{1}{2.3\cdot i_{\mathrm{corr}}}\cdot \frac{b_{a}\cdot b_{c}}{\left(b_{a}+b_{c}\right)}$$

where *i*_corr_ is corrosion current density, *b*_a_ is anodic slope and *b*_c_ is cathodic slope of the alloy.

The corrosion rates (*CR*) were calculated, on the basis of the values of the electrochemical parameters determined from the polarization curve, using Equation (2) according to the ISO G102-89 standard, reapproved in 1999:

(2) $$CR=\frac{K\cdot i_{\mathrm{corr}}\cdot EW}{\rho }$$

where K is a constant for units conversion, *i*_corr_, the corrosion current density of the alloy (µA/cm^2^ or A/cm^2^), *EW* alloy equivalent weight (gram/equivalent), *ρ* alloy density (gram/cm^3^).

#### 2.1.2. Ti25Nb10Zr Alloy Surfaces Functionalization

SAP EAbuK16 was synthesized on solid phase as reported in [49].

Substrates were incubated for 18 h in aqueous solution at different pHs containing 1 mg/mL of EAbuK16. The SAP was dissolved in 10 mM NaCl (Carlo Erba, Cornaredo, Italy) aqueous solutions having different pH values: 0.1 mM HCl (J. T. Baker, Phillisburg, NJ, USA) (pH 4), 0.01 M HCl (pH 2), 0.1 mM NaOH (Carlo Erba) (pH 10), 0.01 M NaOH (pH 12). The pH 7 solution was prepared in two different ways: (a) buffered by Hank’s solution (146.15 mg NaCl; 50 mg KCl (Carlo Erba); 287 mg Na_2_HPO_4_ (Carlo Erba); 50 mg KH_2_PO_4_ (Carlo Erba) in 250 mL distilled water); (b) 10 mM NaCl in distilled water.

The five solutions were then used to cover the alloy surfaces with a layer of SAP. More in detail, thin and thick self-assembling peptide layers namely monolayers, (MLs), and multilayers (MULs), were supported onto Ti25Nb10Zr surfaces as follows:

- MLs: Ti25Nb10Zr discs were sonicated in acetone for 5 min, dried, incubated in the peptide solution for 18 h, washed three times with NaCl 0.10 M at pH 7 and finally three times with distilled water. In these samples the set pH 7 solution was buffered with Hank’s solution to mimic the extracellular physiological environment. Unfortunately, Hank’s solution altered the ionic strength and interfered with peptide deposition. For this reason, the pH 7 sample was prepared again, avoiding the addition of sodium phosphate and other salts, except NaCl 10 mM, and maintaining the 10 mM NaCl washing treatment.
- MULs: Ti25Nb10Zr discs were sonicated in acetone for 5 min and dried. Peptide films were cast by covering the alloy surface with 2–3 drops of 1 mg/mL solutions of EAbuK16 oligopeptide prepared at different pHs, then dried in a low vacuum glass line.

### 2.2. Spectroscopic Techniques

#### 2.2.1. X-ray Photoelectron Spectroscopy

High Resolution X-ray Photoelectron Spectroscopy (XPS) measurements on pristine alloy and multilayer samples were performed at the Materials Science Beamline (MSB) at the Elettra synchrotron radiation source (Trieste, Italy). MSB, placed at the left end of bending magnet 6.1, is equipped with a plane grating monochromator that provides light in the energy range of 21–1000 eV. The UHV endstation, with a base pressure of 1 × 10^−8^ Pa is equipped with a Specs Phoibos 150 hemispherical electron analyzer. Photoelectrons emitted by C1s, N1s, O1s, and Ti2p were detected at normal emission geometry using photon energy of 630 eV, estimated Energy Resolution = 0.6 eV. Binding energies were reported after correction for charging using the aliphatic C1s as a reference (B.E. 285.0 eV). Core-level spectra were fitted with a Shirley background and Gaussian peak functions.

Monolayer samples were investigated at the PM4-LowDosePES beamline at Helmholtz-Zentrum Berlin (BessyII Synchrotron Radiation facility), allowing for a lower flux on the sample, mandatory to avoid damaging the extremely thin layers of peptides. This soft X-ray bending magnet beamline is equipped with a Plane Grating Monochromator operating in collimated light (collimated PGM). It has two permanent end-stations, the reflectometer and the SURICAT (photoemission and X-ray absorption spectroscopy), which are in alternative operation. The LowDose PES end-station is equipped with an SES100 hemispherical analyzer [55]. Energy Resolution was estimated as 0.2 eV.

Conventional XPS studies were performed with an instrument designed by us, consisting of preparation and analysis chambers separated by a gate valve. The analysis chamber is equipped with a six-degree-of freedom manipulator and a 150-mm-mean radius hemispherical electron analyzer with a five-lens output system combined with a 16-channel detector. Ti2p, Nb3d, Zr3d, C1s, O1s, and N1s core level signals were recorded on the investigated samples unmonochromatized MgKα radiation; at least two specimens were analyzed for each sample type. Experimental spectra were analyzed by curve fitting using Gaussian curves as fitting functions; the analyzed spectra were energy referenced to the C1s signal of aliphatic–aromatic C–C carbons located at a binding energy B.E. = 285.0 eV [56,57]. Atomic ratios were calculated from peak areas using Scofield’s cross as section sensitivity factors [58].

#### 2.2.2. Near Edge X-ray Absorption Fine Structure Spectroscopy

Near Edge X-ray Absorption Fine Structure (NEXAFS) spectroscopy experiments were performed at the ELETTRA storage ring at the BEAR (bending magnet for emission absorption and reflectivity) beamline, installed at the left exit of the 8.1 bending magnet exit. The apparatus is based on a bending magnet as a source, a beamline optics delivering photons from 5 eV up to about 1600 eV with a selectable degree of ellipticity. The UHV end station has a movable hemispherical electron analyzer and a set of photodiodes to collect angle resolved photoemission spectra, optical reflectivity, and fluorescence yield, respectively. Moreover, it is equipped with ammeters in order to measure the total electron yield from the sample for NEXAFS measurements [59]. The carbon and nitrogen K-edge spectra were collected at normal (90°), magic (54.7°), and grazing (20°) incidence angles of the linearly polarized photon beam with respect to the sample surface. The photon energy and resolution (Energy Resolution: C K-edge 0.13 eV; N K-edge 0.2 eV) were calibrated and experimentally tested at the K absorption edges of Ar, N_2_, and Ne. In addition, our carbon K-edge spectra were further calibrated using the resonance at 285.50 eV assigned to the C1s π* ring transition. The spectra were then normalized subtracting a straight line that fits the part of the spectrum below the edge and assessing to 1 the value at 320.00 eV and 425.00 eV for carbon and nitrogen, respectively [48].

### 2.3. Microscopy Techniques

#### 2.3.1. Field Emission Scanning Electron Microscopy

(FE-SEM) imaging studies on functionalized Ti25Nb10Zr surfaces were performed at the Charles University (Prague, Czech Republic) as preliminary investigation by means of a HITACHI S-4800 field emission scanning electron microscope operating at 30 keV electron beam energy.

#### 2.3.2. Atomic Force Microscopy

AFM images were recorded on functionalized Ti25Nb10Zr surfaces using an INNOVA microscope (Veeco, Plainview, NY, USA) operating in tapping mode. Each image was acquired on 512 lines with 0.3 Hz on 30 × 30 µm^2^ area. The SPMLab analysis software (Veeco) was used for data processing.

## 3. Results

### 3.1. Characterization of Pristine Ti25Nb10Zr

#### 3.1.1. Assessment of the Elemental Composition and Homogeneity

The composition of the pristine alloy surface was probed by EDS analysis; in Table S1, presented in Supplementary material, the elemental composition determined in four different zones of the alloy surface is shown. It presents the following composition: Ti 68.8 wt %, Nb 21.9 wt %, and Zr 9.3 wt %. No significant differences were observed among the areas, indicating that each element is homogenously distributed on the whole surface. The small differences in EDS alloy composition compared to the value provided by the manufacturer are due to the uncertainty of the investigative techniques. The manufacturing company determined the chemical composition by spark emission spectroscopy. In addition, Figure S1 in the Supporting Information shows the EDS mapping images of the Ti25Nb10Zr alloy performed on the surface corresponding to zone 4 of Table S1. It is evident that each element is evenly distributed over the investigated surface, indicating that the constituent elements are homogenously mixed.

#### 3.1.2. Crystallographic Structure

**Figure 1:**
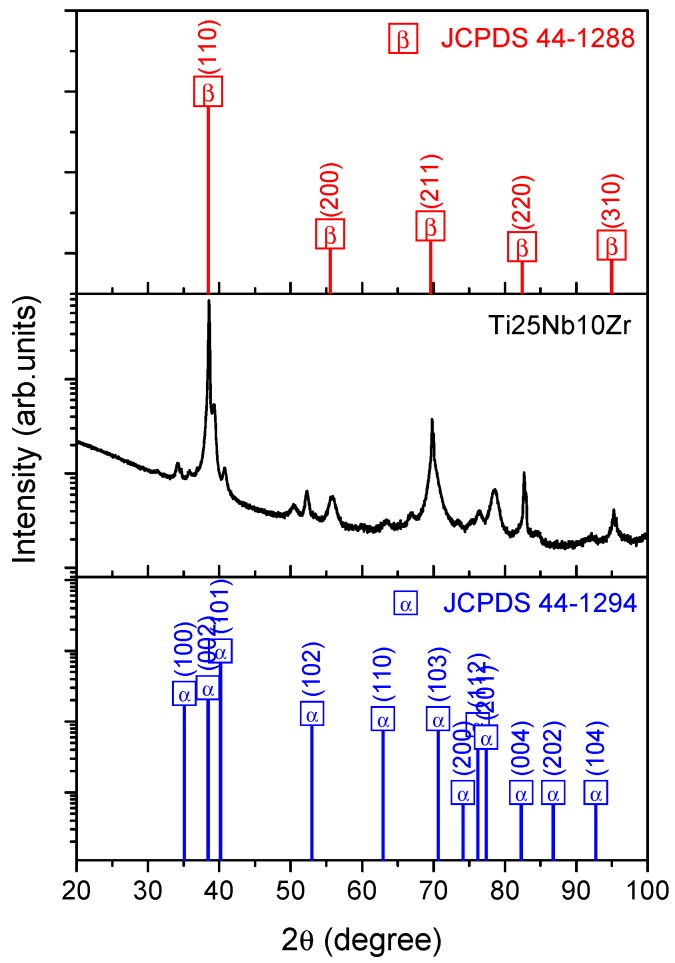
XRD diffraction pattern of Ti25Nb10Zr alloy and JCPDS files No. 44-1294 (α” phase) and 44-1288 (β phase).

The crystallographic structure of the pristine Ti25Nb10Zr alloy was ascertained by means of XRD measurements. The XRD profile is shown in Figure 1. The reflection peaks from both α” (orthorhombic) and β (disordered body-centered cubic) phases were detected. Phases identifications were performed by matching each peak with the JCPDS files No. 44-1284 (α” phase) and 44-1288 (β phase)—Figure 1. Some planes of α” and β phases were overlapped. The α” phase is observed to be the predominant phase. Taking into account the plane of α” phase located at 34.2°, 40.7°, and 52.2°, the grain sizes, calculated by the Scherrer formula, are about 18.7 nm, 15.9 nm, and 18.5 nm respectively, leading to an average of 17.7 nm. In the case of the β phase, taking into account the planes located at 55.7° and 95.4°, which are not overlapped on the α” phase, the grain sizes were calculated to be 8.4 nm and 3.9 nm, with an average of 6.1 nm.

It is noteworthy that no diffraction peak of ω phase (hexagonal structure) was detected. This result is an advantageous one for the proposed alloy, because it is reported in the literature that ω phase causes embrittlement, leading to deterioration of mechanical properties [60,61,62].

#### 3.1.3. Electrochemical Tests

Titanium has a good corrosion resistance in various corrosive environments such as seawater, organic chemicals, oxidizing or reducing acids over a wide range of concentrations and temperatures. This effect is due to oxide forming spontaneously and instantly when its surface is exposed to air and/or corrosive solutions [42]. Ishii et al. reported that the addition of a small amount (up to 3%) of alloying elements in the Ti matrix has a minor effect on the corrosion resistance of Ti in normally passive environments, while under active condition a small amount of alloying elements accelerate the corrosion process, leading to a significant deterioration of the alloy [42]. Thus, when we want to produce novel Ti-based alloys, the effect on its anticorrosive properties of the addition of a new alloying element in the basic matrix is the most important issue in determining whether the Ti passivity is lost and the surface has become fully active.

In the field of the design and production of novel materials for biomedical applications, the high corrosion resistance in physiological solutions is one the most important requirements, because it improves biocompatibility. Thus, for the present study, the Ti25Nb10Zr alloy was tested in terms of its corrosion resistance in two solutions: SBF and Hank at 37 ± 0.4 °C.

**Figure 2:**
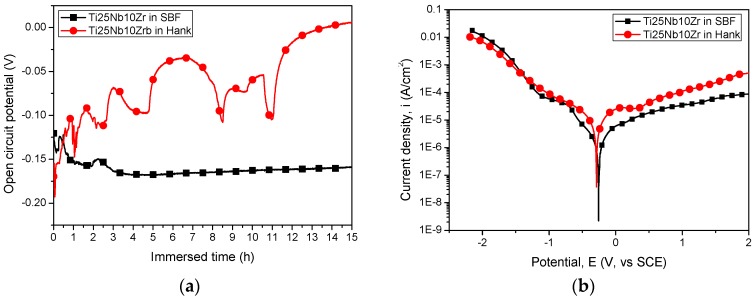
(**a**) Open circuit potential curves of Ti25Nb10Zr alloy in simulated body fluid (SBF) and Hank solutions (symbols are used only for identifying the curves in grey scale printing); (**b**) potentiodynamic polarization curves of Ti25Nb10Zr alloy in SBF and Hank solutions.

The evolution of the open circuit potential (*E*_OCP_) during 15 h of immersion in SBF and Hank solutions at 37 °C is presented in Figure 2a. It is well known that a positive value of *E*_OCP_ indicates that the surface is coved with a protective oxide. In our study, one may see that the alloy immersed in SBF exhibited a more negative value of *E*_OCP_ than that of alloy in Hank solution, but it was stabilized after 4 h of immersion. The evolution of *E*_OCP_ of Ti25Nb10Zr alloy immersed in Hank solution showed many fluctuations, indicating that the oxide is formed and destroyed due to the dissolution and re-passivation processes. This behavior is usually observed for metallic materials immersed in aggressive solutions, due to the presence of aggressive chloride or sulfate ions. Ti25Nb10Zr alloy tested in Hank solution showed some sudden falls of *E*_OCP_, which can be attributed to the metastable pitting corrosion, apparently due to the pit anodic growth. After these falls, the *E*_OCP_ increased, indicating that the surface is re-passivated, by a cathodic oxygen-reduction reaction. This effect was also observed and reported in the literature by Isaacs et al. [63]. So, taking into account the *E*_OCP_ values, we can summarize that the Ti25Nb10Zr alloy is less affected by SBF attack.

The potentiodynamic curves of the Ti25Nb10Zr alloy tested in SBF and Hank solutions at 37 °C are presented in Figure 2b. Based on these curves, the electrochemical parameters were determined, as summarized in Table 1. It is commonly accepted that a surface with a more electropositive corrosion potential, low corrosion current density, and high polarization resistance shows high corrosion behavior. Based on this statement, one may note that Ti25Nb10Zr alloy is more resistant to SBF corrosive attack. This result is also in good agreement with the corrosion rate results. A low corrosion rate was found for the alloy tested in SBF, indicating again that the alloy exhibited good resistance to SBF attack.

In the literature, it is reported that the corrosion rate is an inversely proportional relationship with pH, the corrosion rate decreasing when the pH increases [64]. In our study, the pH of solutions before corrosion tests was 7.4 for both solutions. After the corrosion tests, the pH values were 7.8 and 8.4 for SBF and Hank, respectively, indicating that the alloy is affected more or less by both solutions. In vivo, there is the possibility that the pH exceeds 7.8 in the vicinity of the implant, leading to an alkaline poisoning effect; all the physiological reactions will therefore be unbalanced and a more intense corrosion process will take place with a fast hydrogen evolution process [65]. On this basis, it can be expected that the alloy does not affect the tissue in the vicinity of implant in vivo because is more resistant in SBF. It is important to mention that the Ti25Nb10Zr alloy demonstrated good viability and proliferation after five days of culture with MG63 cells, having values approximately 10% higher than pure Ti [64].

**Figure 3:**
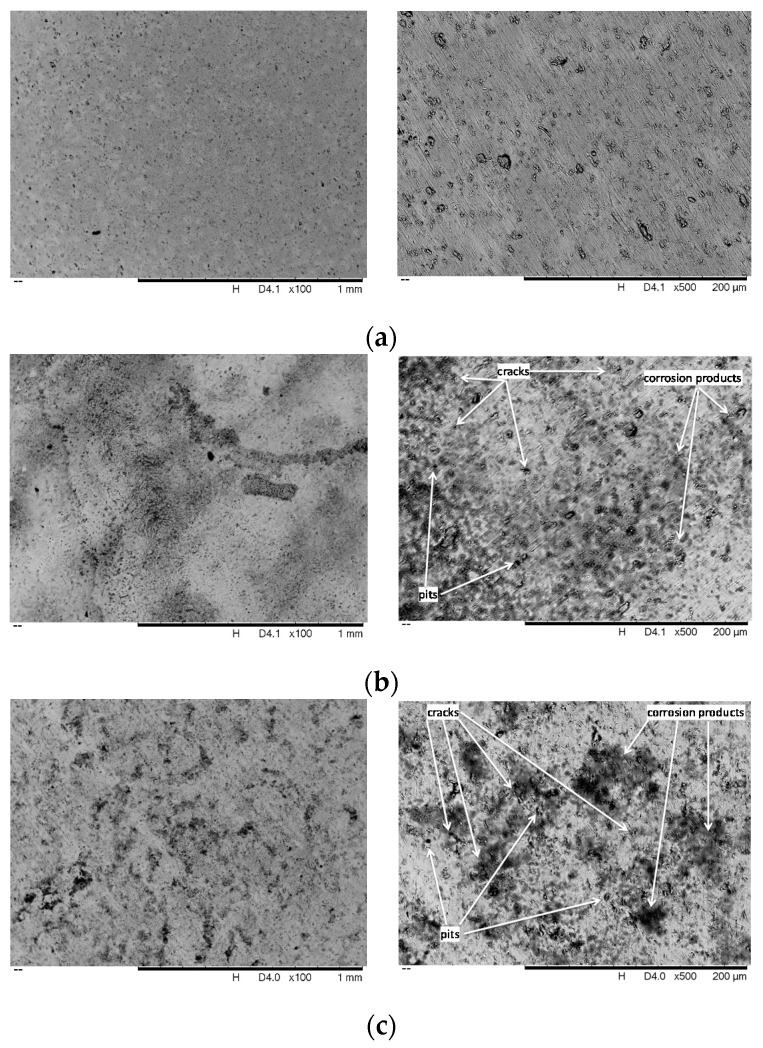
Scanning Electron Microscopy (SEM) images of the alloy surface before and after electrochemical tests. (**a**) Before corrosion; (**b**) after corrosion in SBF; (**c**) after corrosion in Hank.

Figure 3 shows SEM micrographs of the Ti25Nb10Zr alloy before and after corrosion tests at different magnifications. The corrosion tests showed a visible indication of deterioration of alloy in both solutions. No cracks, pores or macro-segregations can be observed on the alloy surface before corrosion (Figure 3a). Some defects can be observed on the alloy surface, probably generated during the polishing process. In both corrosive solutions, the Ti25Nb10Zr alloy suffers a localized corrosion; a relatively uniform attack over the exposed surface of the alloy was found. After corrosion, on the alloy surfaces, pits and cracks can be seen which are the main damage to the surface. Under the Hank testing solution, the alloy corroded more severely compared with alloy tested in SBF solution. Many pits are observed on the alloy tested in Hank solution compared with the SBF one. The localized corrosion increased the current density. According to SEM images, the surface tested in Hank solution is more affected, leading to an increase of current density. This finding is in good agreement with the above potentiodynamic curves. Some grey deposits are found on the alloy surfaces tested in both solutions, which can be related to corrosion products.

The SEM analysis is in good accordance with the open circuit potential measurements and polarization tests.

#### 3.1.4. Surface Composition

Pristine alloy discs were investigated by both Mg K-α source and SR-induced X-ray Photoelectron Spectroscopy (XPS) and with the aim to ascertain the chemical composition at the substrate surface; core level spectra of metal components, i.e., Ti, Nb, and Zr. Ti2p, Nb3d, and Zr3d signals were acquired, confirming the presence of the expected species TiO_2_ (B.E. = 458.35 eV), Nb_2_O_5_ (B.E. = 206.98 eV) and ZrO_2_ (B.E. = 182.10 eV) [56]. The observed atomic ratios Ti 75.4%, Zr 9.4%, Nb 15.2% do not exactly correspond to the expected stoichiometry due to the XPS sensitivity to the sample surface. In fact, since XPS is a surface-sensitive technique, the stoichiometry revealed refers to the outermost layers of the sample surface. Disparity with respect to the expected value could be due to phenomena of interdiffusion towards the outward layers.

**Figure 4:**
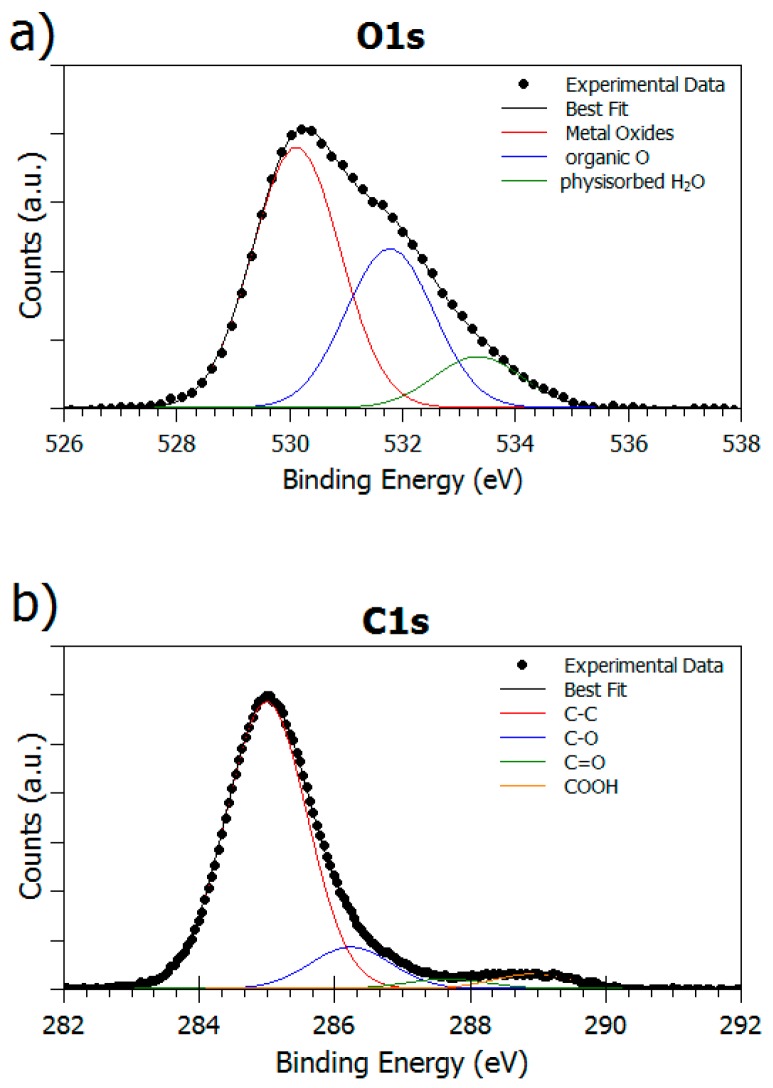
(**a**) XPS O1s and (**b**) XPS C1s spectra of the pristine sample surface.

Since pristine alloy XPS spectra were used as reference for the data analysis of the functionalized samples, O1s, C1s, and N1s core level signal, that are the most indicative for evaluating peptide grafting efficiency at different pH conditions, were also acquired. The O1s signal, reported in Figure 4a, shows a complex structure and, by following a peak-fitting procedure, at least three spectral components can be identified. The first peak at about 530 eV B.E. is attributed to the different metal oxides TiO_2_, Nb_2_O_5_, and ZrO_2_, which we were not able to discriminate, and therefore appear all grouped. At higher BE values a feature due to organic oxygen, arising by contaminating organic matter on the clean alloy surfaces (O–org, B.E. ~ 532 eV) can be singled out; a last component of lower intensity arising by physisorbed water is observed at about 533 eV B.E., as expected from literature for similar samples [65,66]. C1s spectrum is reported in Figure 4b. The curve fitting shows the presence of surface-contaminating carbons, leading to at least four contributions identified as aliphatic (C–C, B.E. = 285,0 eV), single-bonded to oxygen (C–O, B.E. = 286.23 eV), carbonyl-like (C=O, B.E. = 287.63 eV), and carboxyl-like (COOH, B.E. = 288.94 eV) carbons respectively. As expected, the N1s signal is not detected on the surface of “clean” samples.

On the other hand, a complete collection of B.E., Full Width Half Maximum (FWHM), Atomic Ratio values and Feature Assignments for the clean sample is reported in Table S2 in the Supporting Information.

### 3.2. Characterization of Ti25Nb10Zr Surfaces Functionalized with EAbuK16

#### 3.2.1. Analysis of SAP Multilayer (MUL) Adsorbed at Different pH Values

**Figure 5:**
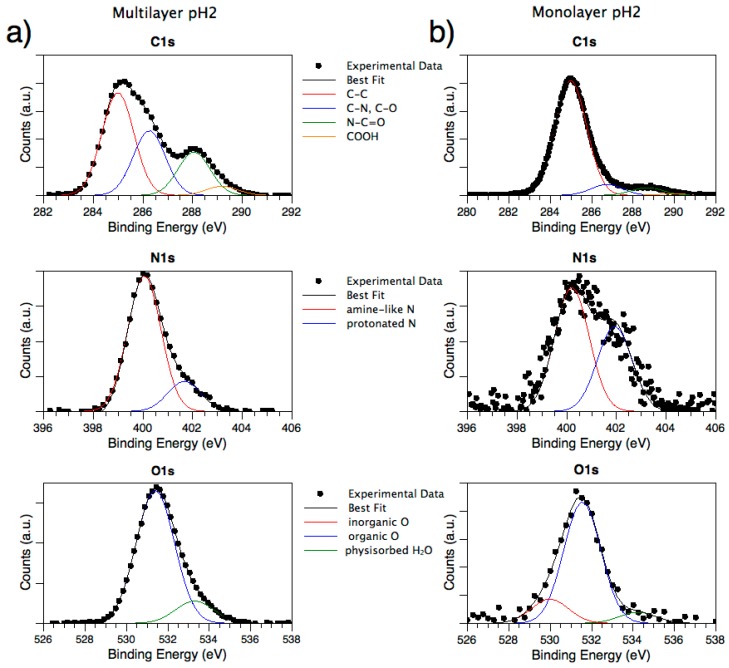
(**a**) X-ray Photoemission Spectroscopy (XPS) C1s, N1s, O1s MUL sample pH 2; (**b**) XPS C1s, N1s, O1s ML sample pH 2.

Samples prepared as MULs from EAbuK16 solutions at pH 2, 4, 7, 10, and 12 were analyzed with both Mg K-α source and SR-induced radiation with the aim to probe molecular stability and anchoring efficiency at different pH values. As shown in Figure 5a for the sample prepared at pH 2 (taken as example), all samples show complex C1s signals (Figure 5a, top spectrum) which were analyzed by the curve-fitting procedure. The main component at lower B.E. values is attributed to aliphatic carbon atoms (C–C, B.E. = 285 eV) and was always used as calibration signal; the peak at about 286.3 eV BE is due to carbons bonded to nitrogens (as expected for aminoacids alpha-carbons, and lysine lateral groups) and/or oxygens; the third component is indicative of peptidic carbons N–C=O (amide-like, B.E. ~ 288.3 eV). The last feature, that can be observed as a small shoulder at higher BE values, is due to glutamic acid carboxyl groups (COOH, B.E. ~ 289 eV) [50,57]. C atoms due to contaminants are also observed in samples functionalized with peptides, giving rise to a signal that is superimposed to the peptide-one, but their amount is comparable in all investigated samples, since they were all treated by following the same procedure.

A complete collection of B.E. and FWHM values is reported in Tables S3 and S4 of Supporting Information. Quantitative estimations of different functional groups are reported in the following Table 2.

By exposing metal substrates to peptide solution, the signals related to nitrogen appear. More in detail, we can discriminate between protonated (B.E. ~ 402 eV) and non-protonated nitrogens (B.E. ~ 400 eV), as expected for peptides layers on metal surfaces, as shown in Figure 5a (spectrum in the middle) [50,57] O1s signals (Figure 5a, bottom) were also collected and compared with the ones observed for the clean substrate. Different from pristine alloy spectra, where all three oxygen species were detected, for MUL samples the signal related to metal oxides is absent, except in pH 7 and pH 12 samples. This indicates that in these two samples the peptide layer is thinner than in others, and since photoelectrons coming from metals are also detected, it is possible to estimate the peptide overlayer thickness by calculating the Ti2p_3/2_ signal’s intensity attenuation in samples with peptide, with respect to the signal intensity in a clean sample, according to equation 3.13 in [58].

Calculated MUL thicknesses for pH 7 and pH 12 are reported in the last column of Table 2.

As for the semiquantitative analysis, C–N/C–C and N–C=O/C–N ratios are more or less constant in all samples, except for sample pH 7, where they are the lowest, as observed in Table 2. This could be due to peptide degradation or non-uniform deposition, as seen in microscopy images, since it has also a greater component related to oxidized carbons (COOH).

#### 3.2.2. Analysis of SAP Monolayer (ML) Adsorbed at Different pH Values

As already observed for MULs, MLs samples (investigated by SR-induced XPS) show complex C1s signals due to the presence of different functional groups, as depicted in Figure 5b for the pH 2 sample. Similar to MULs, B.E. values were calibrated according to C–C aliphatic carbon (285.0 eV); all contributions show the expected value: main peak C–C 285.0 eV B.E., C–N ~ 286.3 eV BE, N–C=O (amide-like) ~ 288.3 eV BE and COOH ~ 289 eV BE. Atomic ratios, reported in Table 3, show a very high amount of aliphatic carbons with respect to MULs, probably due to the higher relative amount of contaminants with respect to the amount of immobilized peptide. However, the relative amounts of C1s spectral components are not influenced by the preparation pH, confirming that the molecular structure of the oligopeptides is always preserved in the anchoring process.

**Figure 6:**
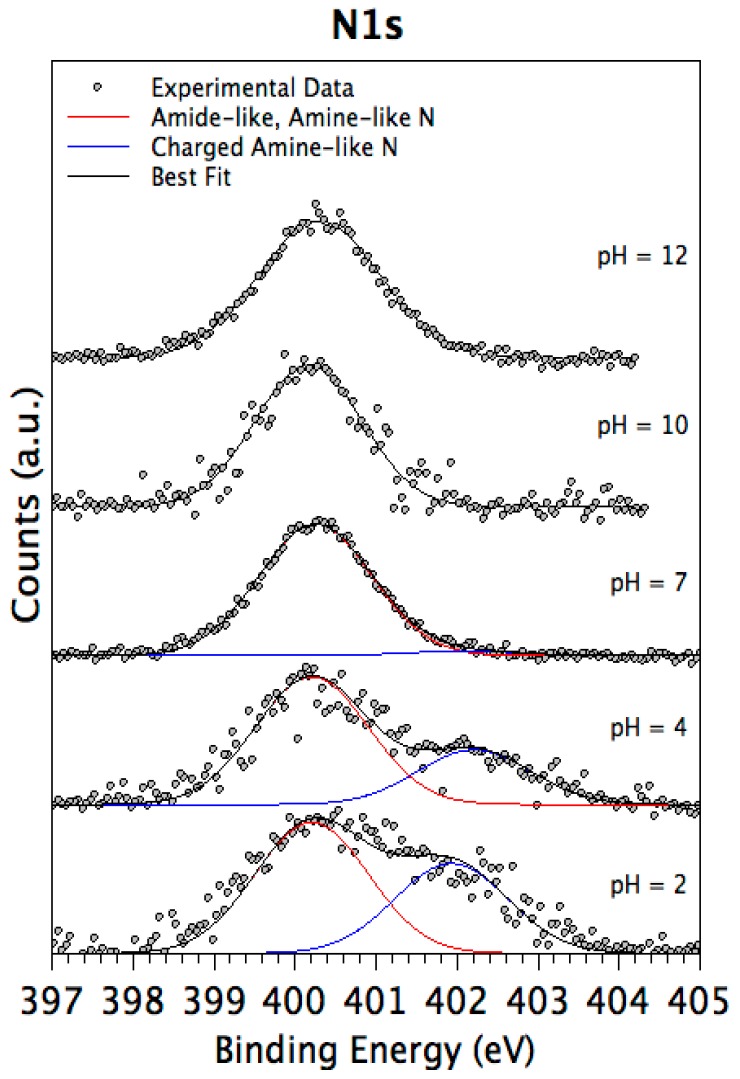
XPS N1s spectra collected on the four monolayer (ML) samples prepared with decreasing pH values.

As expected, all samples present a N1s signal; by applying a peak fitting procedure two main components related to protonated and unprotonated nitrogen atoms can be singled out, as already discussed for MULs. It is noteworthy that the intensity of protonated nitrogen decreases along with the increasing solution alkalinity, as expected (see Table 3). The N1s components trend is clearly observable in the following Figure 6.

**Figure 7:**
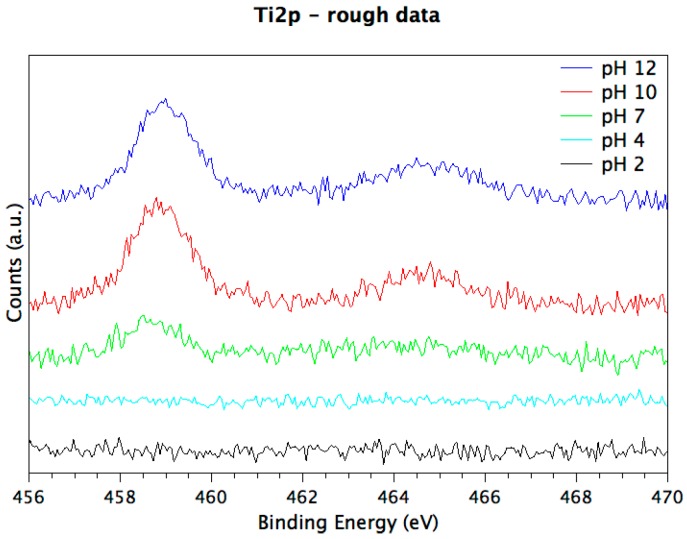
Rough Ti2p spectra, showing the attenuation of substrate signal with decreasing pH, indicative for a better peptide adsorption.

Differently from MUL samples, O1s spectra show all the components observed for pristine samples, i.e., Me–O (~530 eV BE), C–O (~532 eV BE), and physisorbed water (~533 eV BE). The peptide coverage in MLs prepared at neutral and basic pH values (i.e., pH = 7, 10, 12), in fact, is not so thick as to completely screen the substrate signal, as observed in multilayers. This allows the layer thickness to be estimated from the substrate signal attenuation for ML prepared at these three pH values, as reported in Table 3. The trend observed in film thickness is supported by the rough titanium spectra, shown in the following Figure 7.

The observed experimental evidence (C1s components composition preserved in all samples, Ti2p signal attenuation increased with decreasing pH, film thicknesses increased at low pHs, protonated nitrogen component appearing at lower pHs) all concurs to confirm the oligopeptide stability upon anchoring at the titanium alloy surface, and to point out a better surface functionalization performance at low pH values, in good agreement with previous studies carried out on titania surfaces [50,51]. This effect could be related to the net charge of the EAbuK peptide; due to amidation of the carboxyl terminal, the investigated sample has a basic function more than an acidic one and a resulting net charge of +1, increasing peptide solubility at low pH.

#### 3.2.3. Near Edge X-ray Absorption Fine Structure Spectroscopy

NEXAFS (Near Edge X-ray Absorption Fine Structure) is an X-ray absorption technique that, when performed in Angular Dependent mode (i.e., by measuring the X-rays absorption as a function of the incidence angle of the impinging photons with respect to the sample surface), can be usefully applied to evidence whether the peptide molecules are oriented with respect to the surface [51,67]. Data were collected at the C and N K-edges for both MULs and MLs. When the peptide is oriented and organized with respect to the surface a difference in radiation absorption is expected when the angle of the impinging radiation is changed from grazing to normal. The magic incidence is the peculiar angle at which the signal looks as if the peptide was not organized.

**Figure 8:**
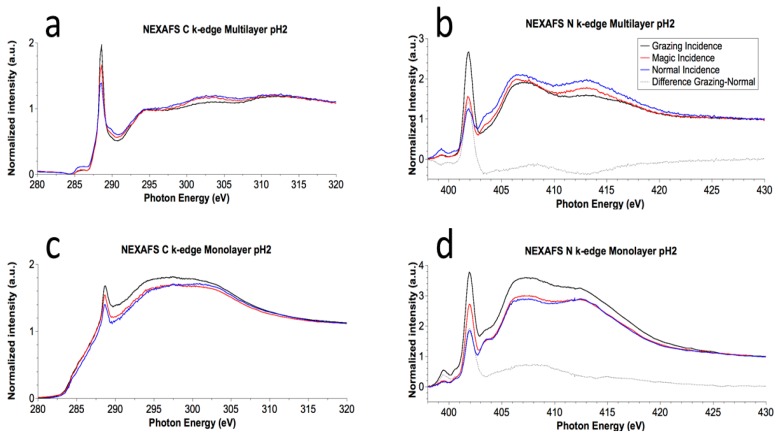
(**a**) C K-edge Near Edge X-ray Absorption Fine Structure (NEXAFS) spectrum of EAbuK multilayer deposited at pH = 2; (**b**) N K-edge NEXAFS spectrum of EAbuK at pH = 2; (**c**) C K-edge NEXAFS spectrum of EAbuK monolayer deposited at pH = 2; (**d**) N K-edge NEXAFS spectrum of EAbuK monolayer at pH = 2.

The carbon spectrum of MUL prepared at pH 2 is reported in the following Figure 8a; in C K-edge spectrum several structures can be observed in both π* and σ* resonances region. The sharp feature at about 288.7 eV is assigned to a C1s → π* transition of C=O molecular orbital, the shoulder around 288 eV to a σ* resonance by the C–H groups, additional features at ≈293 and ≈303 eV can be associated to 1 s → σ* transitions by the C–C and respectively C=O molecular groups.

N K-edge spectra (Figure 8b, MUL pH 2, as an example) show the π* (402 eV) and σ* features (406 eV and ~413 eV) associated with the electronic transitions from N1s to the related antibonding molecular orbitals, as expected from the molecular structure and amino acids sequence [50,51].

The single transition π → π* and the two σ → σ* are represented by the sharp strong peak at 402 eV and 406eV and 412 eV respectively. It is noteworthy how the intensity of transitions π → π* increases from normal to grazing incidence while the intensity of σ → σ* transitions decreases.

ML spectra are completely analogous, as shown in Figure 8c,d for the ML prepared at pH = 2.

The angular dependence analysis evidences changes in the relative intensity for the π* and σ* at different angle resonances in all samples, both MUL and ML, a phenomenon that can be associated with molecular orientation with respect to the surface.

The dichroic behavior of the π* band associated with the peptide bond allowed the tilt angle to be calculated between the π* vector orbital of the peptide bond and normal to the surface, by using the equation reported by Stöhr [67] for threefold or higher symmetry substrates, with a polarization factor *p* = 0.95, and the intensity ratio I_20°_/I_90°_ determined for the selected resonance by peak fitting of the experimental data. For the samples prepared at pH 2, the calculated value for the angle gives rise to a value of the tilt angle between the peptide bond axis (the axis of the main chain) and the substrate surface of nearly 80° (ML θ = 80.2°; MUL θ = 78.2°) for a β-sheet conformation of the peptide backbone. Considering that the incertitude on the tilt angle evaluation is 15% of the calculated value, the two systems have approximately the same molecular orientation.

### 3.3. Microscopy Analysis

#### 3.3.1. FESEM Analysis

**Figure 9:**
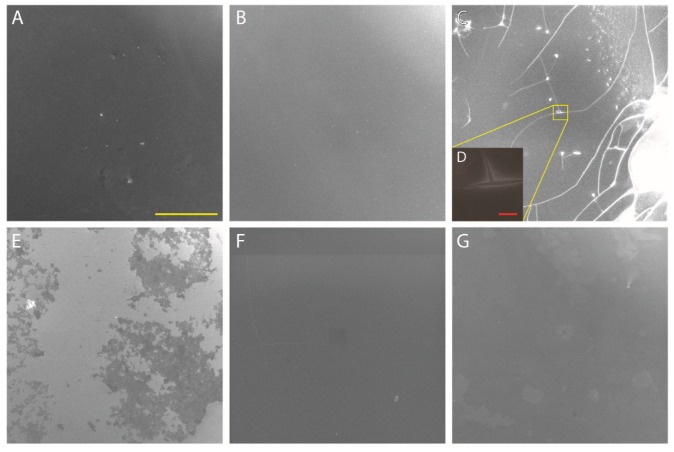
Field Emission Scanning Electron Microscopy (FESEM) images of MUL samples. (**A**): Pristine surface; (**B**): pH 2; (**C**): pH 4; (**D**): magnification of pH 4 region of interest; (**E**): pH 7; (**F**): pH 10; (**G**): pH 12. Yellow bar = 200 μm; Red bar (only D) = 5 μm.

FESEM analysis was performed on MULs in order to investigate whether the peptide aggregates give rise to any peculiar structure. The result is that the peptide covers evenly the surface without assembling in any microscopic or nanoscopic structure detectable with this technique. For all here reported images the yellow bar is 200 μm, except for Figure 9D where the bar is 5 μm. The pristine surface (Figure 9A) appears quite smooth and regular, and peptide immobilization at pH 2, 10 and 12 does not change the appearance (Figure 9B,F,G, respectively). At pH 7 (Figure 9E) the peptide multilayer does not evenly cover the surface, most likely making the layer appear thin at XPS analysis. This result is likely due to the presence of phosphate in solution, in fact the sample prepared without Hank solution is analogous to the other ones. The pH 4 sample (Figure 9C) shows the thickest layer: in fact the peptide surface is extensively cracked because of its thickness. The yellow square identifies the region magnified in Figure 9D, where it is evident how the peptide makes a really thick layer that detaches from the substrate.

#### 3.3.2. AFM Measurements

**Figure 10:**
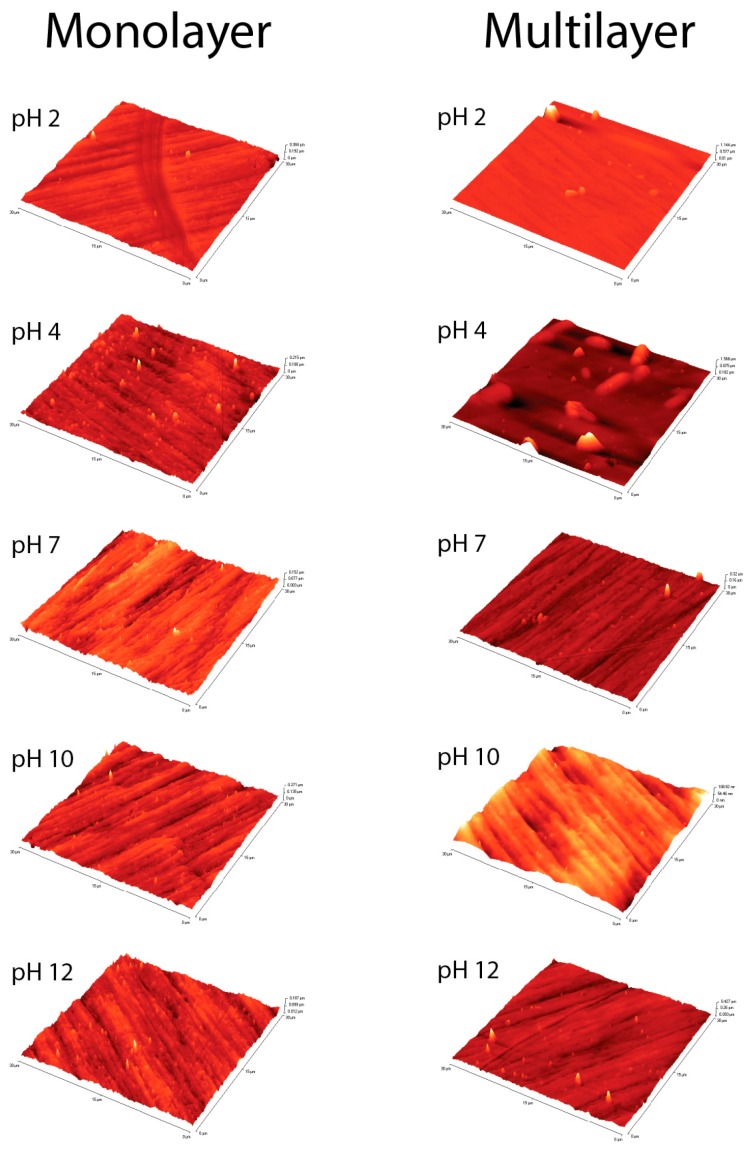
Atomic Force Microscopy (AFM) images of ML and MUL samples. It is evident how surfaces are more uniform at acidic pH. Pristine surface is shown in Supplementary Material Figure S2.

Figure 10 shows AFM images acquired on all samples and on pristine surface. RMS values reported in Table 4 refer to the 900 μm^2^ areas illustrated in Figure 10. It is evident how MUL samples show a more uniform surface with respect to the ML ones, especially at acidic pHs. According to the thickness evaluation reported in Table 3 and Table 4, a thicker layer hides surface irregularity thus establishing an even surface. The pristine surface AFM image is shown in Supplementary Material Figure S2.

## 4. Conclusions

A new titanium alloy for orthopedic prostheses, containing Nb and Zr, was prepared by CCLM technique and characterized by various different techniques. Accurate characterization of the pristine alloy was carried out, revealing homogeneous mixing of the constituent elements (EDS), the presence of both α” (orthorhombic) and β (disordered body-centered cubic) crystalline phases (XRD) and a TiO_2_, Nb_2_O_5_, and ZrO_2_ surface composition (XPS); moreover, electrochemical tests showed that Ti25Nb10Zr alloy corroded more heavily in Hank than in SBF solution, useful information for surface functionalization with peptides. We subsequently investigated the adsorption of EAbuK16, a self-assembling peptide, as a function of pH of the mother solution, both in the monolayer and multilayer. XPS analysis revealed that the amount of adsorbed peptide increases with decreasing pH of the investigated solutions. FESEM and AFM analysis confirmed the formation of a thick and homogenous overlayer at acidic pH. This effect is probably related to the net EAbuK charge of +1, which increases peptide solubility at low pH. NEXAFS investigation yielded evidence of molecular order and orientation of the peptide overlayer with respect to the substrate surface. Formation of an ordered SAP scaffold on the alloy surface should increase osteoblast adhesion to the material surface, thus improving osseo-integration.

## Figures and Tables

**Table 1:** The electrochemical parameters of Ti25Nb10Zr alloy after tests in simulated body fluid (SBF) and Hank solutions (*E*_OCP_—open circuit potential; *R*_p_—polarization resistance; *E*_corr_—corrosion potential at *i* = 0; *i*_corr_—corrosion current density; *CR*—corrosion rate). The *E*_OCP_ values were the last measured values in Figure 2a.

Electrolyte	*E*_OC_ (mV)	*E*_corr_ (mV)	*i*_corr_ (μA/cm^2^)	*R*_p_ (Ω)	*CR* (mm/year)
SBF	−159	−253	1.15	45,011	0.011
Hank	6	−288	14.33	10,676	0.134

**Table 2:** Atomic ratios of the species present on the multilayer (MUL) Self Assembling Peptide (SAP) surface. N^+^: protonated nitrogen; Me–O*_x_*: oxygen of metal oxides; O–org: organic oxygen.

MUL Sample	C–N/C–C Ratio	N–C=O/C–C Ratio	COOH/C–C Ratio	N_tot_/C_tot_ Ratio	N/C_-SAP_ Ratio	N^+^/N_tot_ (%) Ratio	O–org/Me–O*_x_* Ratio	C_-SAP_/Ti Ratio	SAP Thickness (nm)
pH 2	0.63	0.42	0.09	0.18	0.90	18.11	----	----	----
pH 4	0.57	0.37	0.05	0.14	0.77	15.57	----	----	----
pH 7	0.37	0.13	0.17	0.02	0.28	0.00	1.29	4.07	1.54
pH 10	0.56	0.37	0.06	0.16	0.87	10.99	----	----	----
pH 12	0.48	0.27	0.10	0.11	0.76	11.33	1.75	8.17	4.31

**Table 3:** Atomic ratios of the species present on ML. N^+^: protonated nitrogen; Me–O*_x_*: oxygen of metal oxides; O–org: organic oxygen.

ML Sample	C–N/C–C Ratio	N–C=O/C–C Ratio	COOH/C–C Ratio	N_tot_/C_tot_ Ratio	N/C_-SAP_ Ratio	N^+^/N_tot_ (%) Ratio	O–org /Me–O*_x_* Ratio	C_-SAP_/Ti Ratio	SAP Thickness (nm)
pH 2	0.44	0.25	0.10	0.11	0.77	13.47	24.76	2.91	1.22
pH 4	0.42	0.21	0.12	0.06	0.23	9.20	1.83	13.41	3.10
pH 7	0.34	0.12	0.10	0.04	0.58	4.13	1.04	1.76	1.28
pH 10	0.38	0.19	0.12	0.05	0.46	0.00	1.67	4.07	2.52
pH 12	0.32	0.16	0.11	0.04	0.42	0.00	0.96	2.15	1.32

**Table 4:** Root mean square (RMS) roughness values for multilayer samples (MUL) and monolayer samples (ML).

Sample	RMS (µm)	
Clean Surface	0.0143	
	MUL RMS (µm)	ML RMS (µm)
pH 2	0.0181	0.0446
pH 4	0.0136	0.1067
pH 7	0.0155	0.0136
pH 10	0.0201	0.0141
pH 12	0.0127	0.0174

## Supplementary Materials

The following are available online at http://www.mdpi.com/2079-4991/8/3/148/s1, Figure S1: Total EDS mapping spectrum and relative distribution of the concentration of each constituent element on the Ti25Nb10Zr alloy surface performed in a one scanned zone on the surface, Figure S2: AFM image of Clean Surface Sample, Table S1: The elemental composition of the alloy determined by EDS measurements, Table S2: Binding Energies in eV of the atomic species present on the Clean Surface Sample and the relative atomic ratios, Table S3: Binding Energies in eV of the atomic species present on the MUL sample surface, Table S4: Binding Energies in eV of the atomic species present on the ML sample surface.
