# Supplementary material for: Biocompatible Materials Based on Self-Assembling Peptides on Ti25Nb10Zr Alloy: Molecular Structure and Organization Investigated by Synchrotron Radiation Induced Techniques
Source: Nanomaterials (Basel). 2018 Mar 7;8(3):148. doi: 10.3390/nano8030148 (PMC5869639; doi:10.3390/nano8030148)

Biocompatible Materials Based on Self-Assembling Peptides on Ti25Nb10Zr Alloy: Molecular Structure and Organization Investigated by Synchrotron Radiation Induced Techniques.

Valeria Secchi [1], Stefano Franchi [1,*,†], Marta Santi [1], Alina Vladescu [2], Mariana Braic [2], Tomáš Skála [3], Jaroslava Nováková [3], Monica Dettin [4], Annj Zamuner [4], Giovanna Iucci [1] and Chiara Battocchio [1,*]

1. Department of Science, Roma Tre University of Rome, Via della Vasca Navale 79, 00146 Rome, Italy; valeria.secchi@uniroma3.it (V.S.); santimarta3@gmail.com (M.S.); giovanna.iucci@uniroma3.it (G.I.)
2. National Institute for Optoelectronics, 409 Atomistilor St., 077125 Magurele, Romania; alinava@inoe.ro (A.V.); mariana.braic@inoe.ro (M.B.)
3. Department of Surface and Plasma Science, Faculty of Mathematics and Physics, Charles University, V Holešovičkách 2, 18000 Prague, Czech Republic; tomas.skala@elettra.eu (T.S.); jaroslava.lavkova@gmail.com (J.N.)
4. Department of Industrial Engineering, University of Padua, Via Marzolo, 9, Padua 35131, Italy; monica.dettin@unipd.it (M.D.); annj.zamuner@studenti.unipd.it (A.Z.)

* Correspondence: stefano.franchi@elettra.eu (S.F.); chiara.battocchio@uniroma3.it (C.B.); Tel.: +39-040-3758059 (S.F.); +39-06-5733-3400 (C.B.)

† Present Address: Elettra-Sincrotrone Trieste S.C.p.A. di interesse nazionale, Strada Statale 14-km 163,5 in AREA Science Park, 34149 Basovizza, Trieste, Italy.

Supplementary Material

Figure S1. Total EDS mapping spectrum and relative distribution of the concentration of each constituent element on the Ti25Nb10Zr alloy surface performed in one scanned zone on the surface (zone 4 of Table S1)

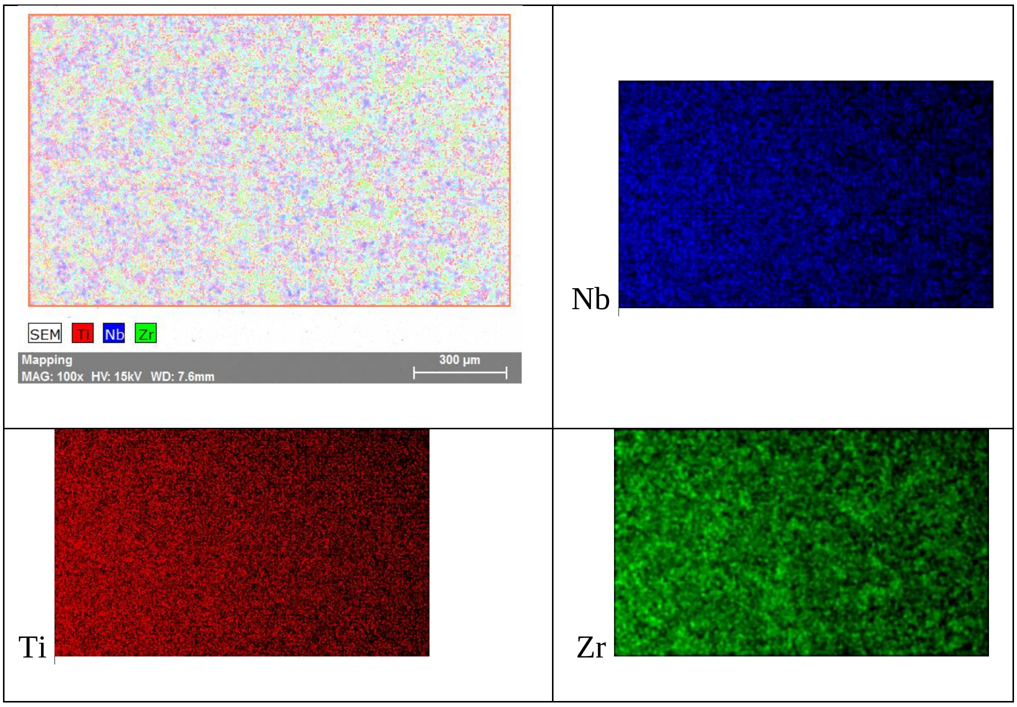

Table S1. The elemental composition of the alloy determined by EDS measurements

Zone	Ti (wt%)	Nb(wt %)	Zr (wt %)
Zone 1	68.7	21.9	9.4
Zone 2	69.2	21.6	9.2
Zone 3	68.7	22.1	9.2
Zone 4	68.8	21.8	9.4
Average	68.85±0.24	21.85±0.21	9.3±0.11

Table S2. Binding Energies in eV of the atomic species present on Clean Surface Sample and relative atomic ratios. Me-Ox: oxygen of metal oxides;
O-org: organic oxygen.

	C1s				O1s					
	C-C	C-O	C=O	COOH	Me-Ox	O-org	H_2O	$Ti2p_{3/2}$	$Nb3d_{5/2}$	$Zr3d_{5/2}$
B.E. (eV)	285.0	286.4	287.9	288.1	530.1	531. 8	533.3	458.5	207.1	182.2
FWHM (eV)	1.6	1.6	1.6	1.6	1.8	1.8	1.8	1.8	1.7	1.6
		C-O/ C-C	C=O/ C-C	COOH/ C-C		O org/ TiO_2	C tot /Ti	Ti/Nb	Ti/Zr	Nb/Zr
Atomic Ratio (%)		31	12	13		61	676	380	522	159

Table S3. Binding Energies in eV of the atomic species present on MUL samples surface. N+: protonated nitrogen; Me-Ox: oxygen of metal oxides; O-org: organic oxygen; ----: not detected.

	C1s				N1s		O1s					
MUL sample	C-C (eV)	C-N (eV)	N-C=O (eV)	COOH (eV)	N (eV)	N+ (eV)	M-Ox (eV)	O-org (eV)	H_2O (eV)	$Ti2p_{3/2}$ (eV)	$Nb3d_{5/2}$ (eV)	$Zr3d_{5/2}$ (eV)
pH 2	285.0	286.3	288.1	289.1	400.1	401. 7		531.4	533.3			
FWHM	1.5	1.5	1.5	1.5	1.6	1.6		2.2	2.2			
pH 4	285.0	286.3	288.3	289.5	400.2	401.8		531.7	533.5			
FWHM	1.6	1.6	1.6	1.6	1.6	1.6		2.1	2.1			
pH 7	285.0	286.3	287.6	289.0	401.3		530.8	532.7	534.2	459.5	208.2	
FWHM	1.6	1.6	1.6	1.6	1.9		2.0	2.0	2.0	2.3	1.9	
pH 10	285.0	286.3	288.2	289.3	400.1	401.8		531.2	533.0			
FWHM	1.6	1.6	1.6	1.6	1.6	1.6		2.1	2.1			
pH 12	285.0	286.3	288.1	289.1	400.2	401.9	530.8	532.6	534.6	458.5	207.1	182.1
FWHM	1.6	1.6	1.6	1.6	1.6	1.6	2.3	2.3	2.3	1.9	2.0	2.1

Table S4. Binding Energies in eV of the atomic species present on ML sample surface. N+: protonated nitrogen; Me-Ox: oxygen of metal oxides; O-org: organic oxygen.

	C1s				N1s		O1s					
ML Sample	C-C (eV)	C-N (eV)	N-C=O (eV)	COOH (eV)	N (eV)	N+ (eV)	M-O_x (eV)	O-org (eV)	H_2O (eV)	Ti 2p 3/2 (eV)	Nb 3d 5/2 (eV)	Zr 3d 5/2 (eV)
pH 2	255.0	286.4	288.1	289.2	400.2	401.8	530.2	531.7	533.3	458.8	207.1	182.2
FWHM	1.6	1.6	1.6	1.6	1.6	1.6	1.9	1.9	1.9	1.9	1.6	1.4
pH 4	285.0	288.0	286.3	289.1	400.0	401.4	530.3	531.6	533.2	458.5	207.0	182.1
FWHM	1.5	1.5	1.5	1.5	1.5	1.5	2.1	2.1	2.1	1.7	2.3	2.1
pH 7	285.0	286.3	287.3	289.0	400.1	401.4	530.3	531.9	533.5	458.6	207.3	182.3
FWHM	1.6	1.6	1.6	1.6	1.7	1.7	2.0	2.0	2.0	1.9	1.7	1.7
pH 10	285.0	286.3	288.0	289.2	400.2		530.4	531.7	533.3	458.7	207.3	182.4
FWHM	1.6	1.6	1.6	1.6	1.8		2.1	2.1	2.1	1.8	1.5	1.8
pH 12	285.0	286.4	287.9	289.1	400.3		530.6	532.1	533.7	458.7	207.4	182.5
FWHM	1.6	1.6	1.6	1.6	1.8		2.0	2.0	2.0	1.7	1.7	2.0

Figure S2. AFM image of Clean Surface Sample

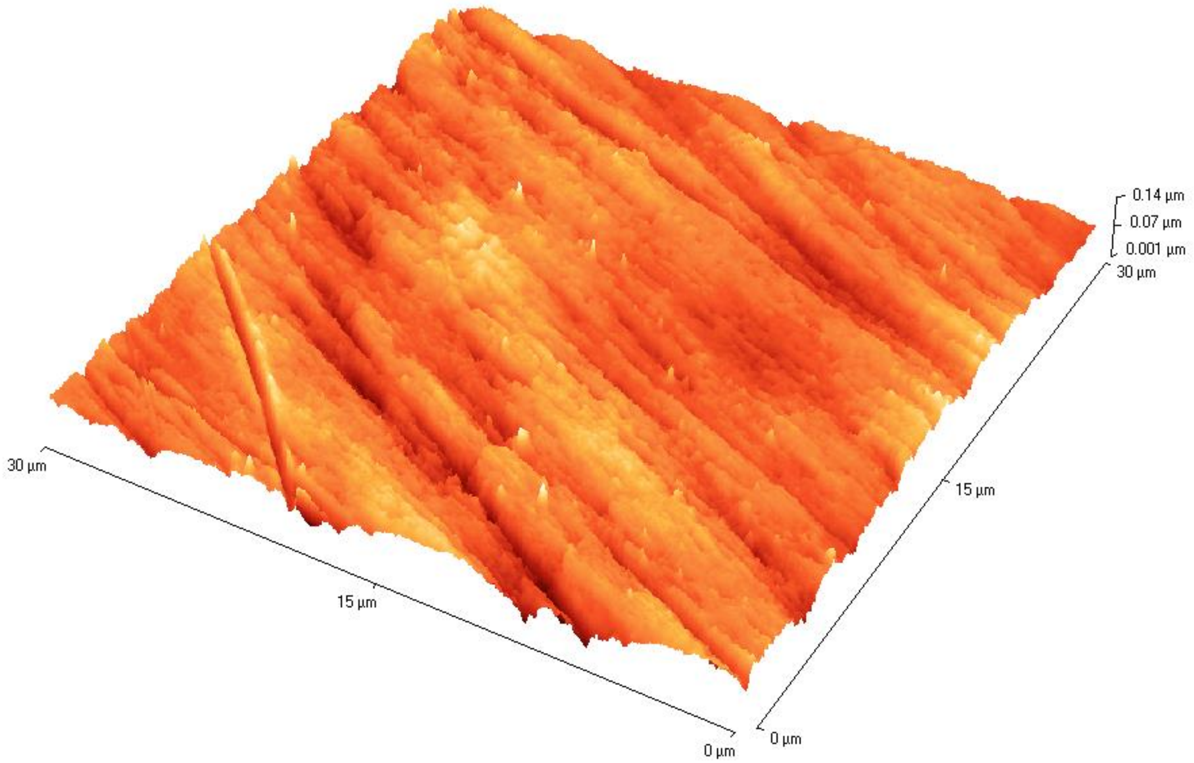

Supplement: Supplementary file 1 [file nanomaterials-08-00148-s001.pdf]
